# High Glucose Induces Biomolecular Dysregulation in Primary Human Corneal Epithelial Cells: Potential Biomarkers of Hyperglycemia-Driven Corneal Impairment

**DOI:** 10.3390/ijms27188406

**Published:** 2026-09-21

**Authors:** Lucia Dinice, Bijorn Omar Balzamino, Graziana Esposito, Rosanna Squitti, Antonio Di Zazzo, Alessandra Micera

**Affiliations:** 1 Research and Development Laboratory for Biochemical, Molecular and Cellular Applications in Ophthalmological Science, IRCCS—Fondazione Bietti, 00184 Rome, Italy; lucia.dinice@fondazionebietti.it (L.D.); bijorn.balzamino@fondazionebietti.it (B.O.B.); graziana.esposito@fondazionebietti.it (G.E.); 2 Department of Laboratory Science, Research and Development Division, Ospedale Isola Tiberina—Gemelli Isola, 00186 Rome, Italy; rosanna.squitti@fbf-isola.it or rosanna.squitti@ecampus.it; 3 Department of Theoretical and Applied Sciences (DISTA), eCampus University, 22100 Novedrate, CO, Italy; 4 Ophthalmology Unit, University Campus Bio-Medico, 00128 Rome, Italy; a.dizazzo@policlinicocampus.it; 5 Corneal Rare Disease Center, Ophthalmology, Campus Bio-Medico University Hospital Foundation, 00128 Rome, Italy

**Keywords:** hyperglycemia, ocular surface, cornea, diabetes, biomarkers, innate immunity, complement, *TLR-2*, inflammation, complement factor H

## Abstract

Corneal hyperglycemia is associated with epithelial dysfunction characterized by impaired inflammation, oxidative stress, neuropathy and innate immune mechanisms, as observed in diabetic corneal keratopathy. Therefore, the aim of the study was to investigate at cellular level the effects of moderate to severe acute and chronic challenges of glucose on human primary cultures of corneal epithelial cells (CECs). Human CECs (Innoprot, Spain) were exposed to single or repeated 15 mM or 30 mM High Glucose (HG)-supplemented media (CEC Medium containing plain 5 mM glucose), every 2 days from first stimulations and harvested at day 1 (single/acute challenge) or at days 3 and 5 (repeated/chronic challenge). Mannitol was used as osmotic control and cell sustainability were monitored by trypan blue exclusion test and MTT. Conditioned media were subjected to protein analysis while monolayers were processed for relative real-time RT-PCR. Few selected mediators belonging to the inflammatory, complement, innate receptor, oxidative, DNA enzymes and neurotrophic pathways were tested, and protein levels were assayed on selected mediators (IL-6, IL-8, CFH and C5b-9; ELISA). HG exposure induced a time- and concentration-dependent decrease in CEC metabolic activity, without a comparable reduction in cell number. Acute HG exposure promoted an inflammatory response characterized by increased *p65NF-κB*, *IL-1β*, *IL-6* and *IL-8* transcripts, together with an early modulation of complement components and increased expression of *CD55* and *CD59* (regulators). Chronic HG exposure induced a marked shift toward complement activation, with increased *C3aR1*, *C5aR1*, *C3*, *C5* and *C9* transcripts and reduced *CD55* and *CD59* expression, with a progressive accumulation of CFH and C5b-9 proteins in a positive relationship. Chronic HG also increased IL-6 and IL-8 protein release and induced a coordinated upregulation of *C5*, *C5aR1* and *TLR-2*, particularly at 30 mM HG. Antioxidant, DNA enzymes and neurotrophic pathways were differentially modulated over time, with an early *Nrf-2* response followed by chronic *Keap-1* upregulation and *Nrf-2* reduction, progressive *DNMT3a*/*HDAC1* expression, and a biphasic *β-NGF*/*BDNF* transcriptional profile. Overall, these findings support a proposed biphasic model of the CEC response to hyperglycemia, characterized by an initial adaptive molecular response followed by progressive dysregulation under prolonged exposure. This early molecular profile might represent an adaptive response to acute metabolic stress, although further studies are required to confirm this functional protective significance.

## 1. Introduction

The corneal epithelium is the outermost cellular layer of the eye, serving as a primary barrier against environmental insults and as an active participant in innate immune surveillance [1]. Given its crucial role in ocular defense, any compromise to this functional unit can predispose the cornea to infections affecting the refractive capabilities [2].

At the ocular surface, metabolic diabetes triggers a cascade of morphological and functional alterations, affecting corneal nerve and epithelial integrity and developing dry-eye discomfort or disease [3]. This hyperglycemia-induced damage on the corneal surface is multifactorial and undervalued. At the cellular level, chronic High Glucose (HG) exposure drives primary mitochondrial dysfunction, leading to the overproduction of reactive oxygen species (ROS) that in combination with advanced glycation end-products (AGEs) triggers neurotrophic deficits, axonal degeneration and endothelial dysfunction, compromising the ocular surface purpose [4,5,6]. Alongside metabolic and oxidative mechanisms, innate immune response, complement and neurotrophic participation have emerged as additional layers of pathological response in diabetic-target organs [7].

Complement fragments and receptors belonging to the innate immunity (Toll-like receptors; TLRs) work in concert to guarantee host defense that is compromised/dysregulated by diabetes, becoming detrimental and driving systemic inflammation [8,9]. Complement dysregulation has been documented in diabetes multiple-target organs and particularly hyperglycemia showed complement activation through AGE-mediated pathway induction, followed by downregulation of membrane-bound regulatory proteins and impaired fluid-phase inhibition, all mechanisms primarily characterized at the retina and kidney, but still underexplored at the corneal epithelium [10,11]. Particularly, the complement system is involved in the occurrence and development of diabetic retinopathy through all major complement pathways, with C3a, C5a, and the membrane attack complex contributing to pathological damage either through direct damaging effects or by activating cells in the retinal microenvironment [10]. Previous studies using immortalized cell lines demonstrated that High Glucose could suppress *TLR-2*/*TLR-4* expression, potentially impairing the innate immune response to pathogens, as commonly observed in diabetic subjects [12].

Therefore, while oxidative stress and growth factor dysregulation have been extensively characterized in diabetic cornea, the contribution of some pro-inflammatory, complement and innate immune signaling to the hyperglycemia-driven transcriptional response of corneal epithelial cells remains poorly understood [3].

Herein we sought to investigate the time and dose-dependent effects played by High Glucose (HG) on primary corneal epithelial cells (CECs) cultured under single and harvesting at day 1 (acute challenge), or under multiple HG exposures (every 2 days) with harvesting at day 3 or day 5 (chronic challenge).

## 2. Results

The appropriate in vitro High Glucose (HG) concentration was assessed starting from literature and evaluated in preliminary studies [13,14].

**Figure 1:**
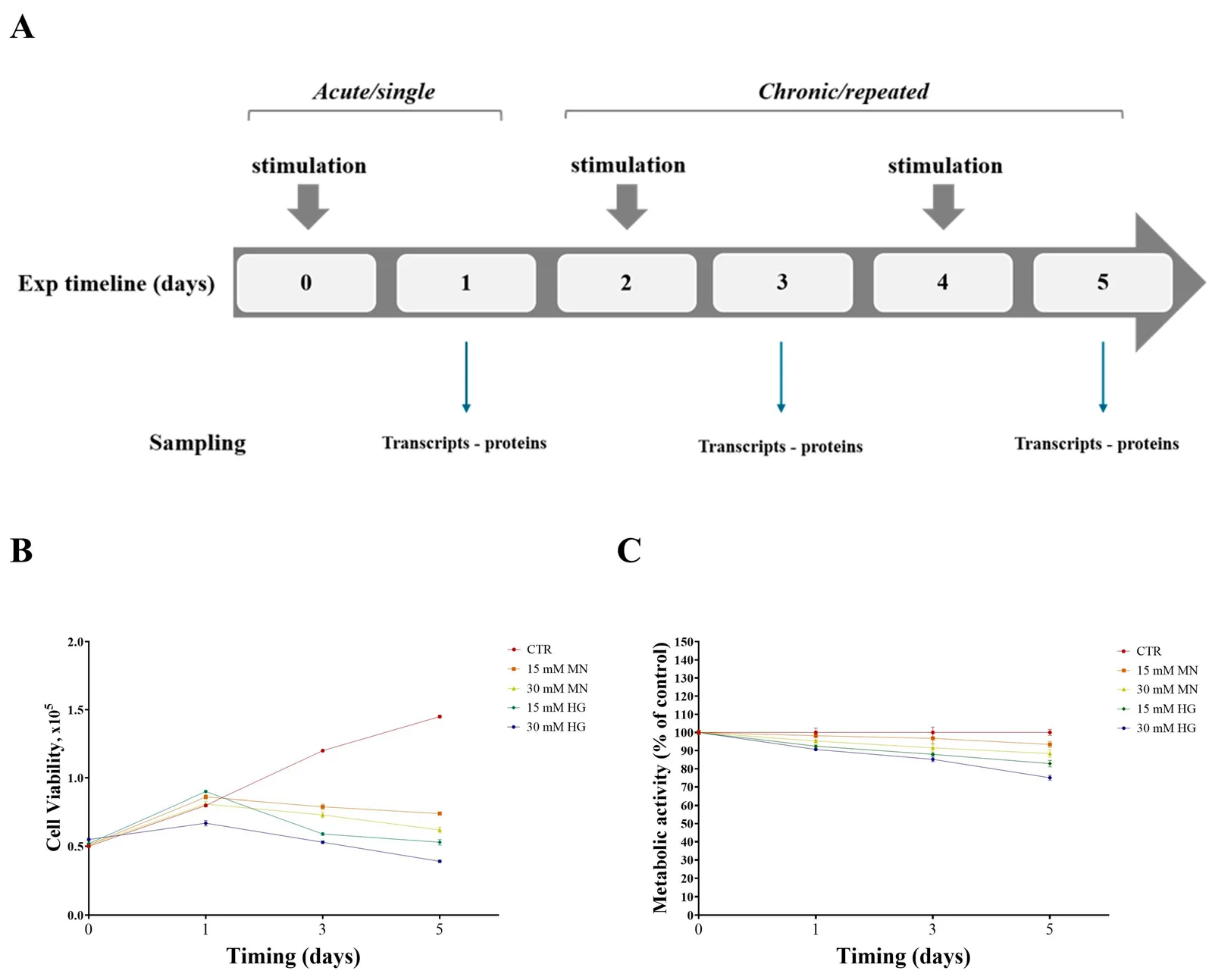
Experimental procedure and cell behavior. (**A**) Timeline of experimental procedure reporting stimulations and sampling tasks, considering acute (single) and chronic (repeated) exposure conditions (gray arrows). Stimulations were performed every 2 days (0, 2, 4) with sampling 24 h after the last stimulation. Conditioned media and monolayers sampled on day 1, 3, 5, for transcript and protein analyses (blue arrows). (**B**) Line-graph over time showing the cell viability expressed as total number of viable cells (×10^5^) for all conditions, according to the trypan blue technique. (**C**) Line-graph over time showing the metabolic activity upon stimulation as calculated by the formula [(MTT treated/MTT control) × 100] obtained after MTT technique. Data are expressed as mean % ± SEM from four independent experiments carried out in triplicate. Legend: CTR, normal glucose; HG, High Glucose; MN, Mannitol.

To establish the experimental conditions for acute and chronic exposure in vitro, monolayers were exposed to a supplementation of 15 mM or 30 mM glucose every two days (baseline/day 0, day 2 and day 4) and appropriate samplings (conditioned media and cell harvesting) were carried out every 24 h from the last stimulation (day 1, day 3, day 5), mimicking a single (day 1; acute challenge) and repeated (day 3 and day 5; chronic challenge) exposure, as visible in the experimental timeline (Figure 1A). Molecular (transcripts) and biochemical (proteins) analyses performed at day 1, day 3 and day 5 (Figure 1A). The same experimental protocols were carried out in preliminary experiments with Mannitol (MN), to assess the impact of sustained osmotic exposure.

Cell viability (Figure 1B) and metabolic activity (Figure 1C) were monitored throughout the experimental timeline to assess the impact of sustained HG exposure.

As shown in Figure 1B, all experimental groups displayed comparable cell numbers at baseline (day 0; plating 0.5 × 10^5^ cells/well). Upon stimulation, cell viability increased at day 1 in all stimulated conditions although was not maintained during repeated exposure. From day 1 to day 3, cell viability progressively decreased under both glucose and mannitol conditions, with the most pronounced reduction observed with 30 mM HG (0.45 × 10^5^ cells/well). At day 5, cell viability in 30 mM HG group was markedly lower than that observed in control cells. The control group (5 mM glucose/medium) showed a progressive increase in cell number over time (day 5; 1.45 × 10^5^ cells/well). Regarding the metabolic activity (Figure 1C), while control cells maintained metabolic activity close to baseline throughout the experimental period, both 15 mM and 30 mM HG conditions triggered a time- and dose-dependent decrease that was mainly evident under 30 mM HG, progressively reaching approximately 75% of control levels (day 5). On the contrary, cells exposed to 15 mM HG showed a milder decrease, remaining at approximately 85–90% of control values at day 5.

For molecular analysis, the biomarkers were a few selected candidates belonging to inflammatory, complement, recognition receptors, oxidative stress/redox, DNA enzymes and neurotrophic pathways. For biochemical analysis, production and release of inflammatory IL-6 and IL-8 cytokines and complement regulator CFH were quantified in conditioned media (ELISA) while C5b-9 protein complex was measured in cellular extracts and conditioned media.

### 2.1. HG Influenced the Transcript Expression of Pro-Inflammatory Mediators Under Acute and Chronic Challenges

**Figure 2:**
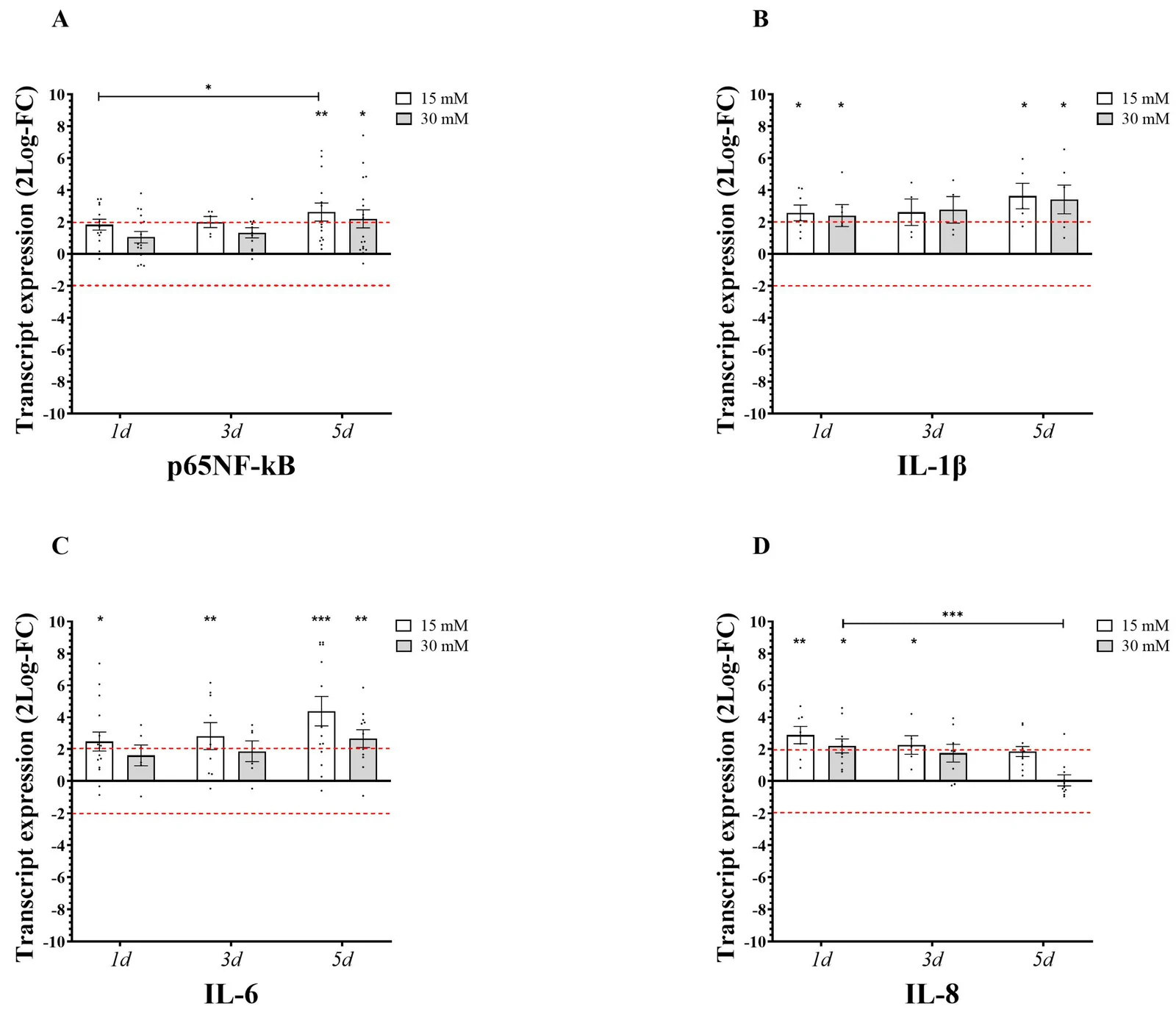
HG-exposed CECs display differential transcript expressions of pro-inflammatory mediators. Bar graphs showing the transcript expression ratios (2 log-fold changes) specific for *p65NFκB* (**A**), *IL-1β* (**B**), *IL-6* (**C**) and *IL-8* (**D**) in CECs exposed to 15 mM HG (white bars) or 30 mM HG (grey bars) over 1, 3 and 5 days. Expression ratios were calculated relative to untreated control cells. Data are expressed as mean ± SEM from four independent experiments carried out in triplicate. The red dashed lines were included as an additional stricter cutoff identifying biologically relevant changes. Data was analyzed by two-way ANOVA and mixed-effect models with Dunnett’s post hoc tests for time points with respect to related untreated groups and between groups. Statistical significance was set at * *p* < 0.05; ** *p* < 0.01; *** *p* < 0.001. Legend: *p65NFκB*, Nuclear Factor kappa B subunit p65; *IL*, Interleukin.

To characterize the inflammatory response of CECs exposed to single and chronic HG exposure, we assessed the transcript expression of few well-recognized pro-inflammatory mediators of the ocular surface *(p65NF-κB*, *IL-1β*, *IL-6* and *IL-8*) (Figure 2). Merely, *p65NF-κB* mRNA (Figure 2A) showed a trend to an increase overtime for both doses, peaking at 30 mM HG on day 5. As well, *IL-1β* mRNA expression (Figure 2B) was higher than 2 Log-FC across all conditions over timing, although the higher transcript expressions were monitored at 5 days for both 15 mM and 30 mM HG, with no difference between concentrations. Likewise, *IL-6* mRNA expression (Figure 2C) increased in a time-dependent manner, peaking at 15 mM HG with respect to 30 mM HG. Finally, *IL-8* mRNA expression (Figure 2D) was higher at 1 day for both concentrations and decreased in a time-dependent fashion reaching the lower expression at 15 mM and unchanged values at 30 mM HG with respect to controls.

**Figure 3:**
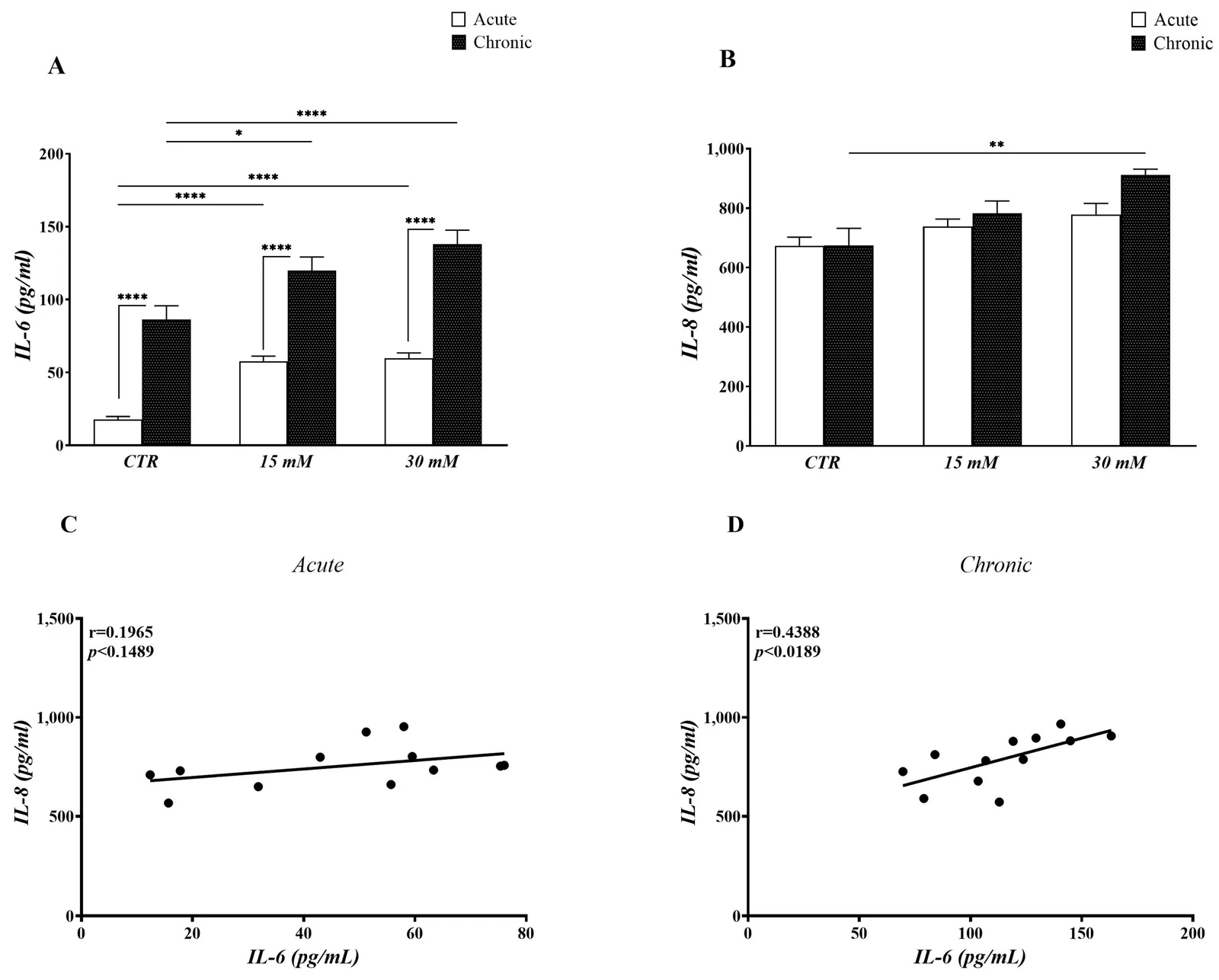
HG-exposed CECs display differential IL-6 and IL-8 protein levels in the conditioned media. IL-6 (**A**) and IL-8 (**B**) protein levels were measured (ELISA) in conditioned media and compared for acute (1 day) and chronic (5 days) stimulation, and between doses. Note the huge IL-6 protein level between acute and chronic exposure at every dose tested 30 mM higher than IL-6 ones. Data are expressed as mean ± SEM from four independent experiments (pg/mL). Two-way ANOVA followed by a Tukey–Kramer multiple comparisons test and asterisks indicate the observed statistical significance (* *p* < 0.05; ** *p* < 0.01; **** *p* < 0.0001). (**C**,**D**) Scatterplots display a positive relationship between IL-6 and IL-8 upon acute (**C**) and chronic (**D**) HG exposure. Pearson correlation coefficient (r) and significance are shown in the panel. Legend: IL, Interleukin.

According to their significant difference between 15 mM and 30 mM HG exposure at 5 days, IL-6 and IL-8 were further investigated with respect to protein release into conditioned media, as quantified by ELISA. As shown in Figure 3A, conditioned media from CECs exposed to normal glucose (5 mM), 15 mM HG and 30 mM HG showed higher IL-6 protein levels when chronically stimulated. The same results were observed for IL-8 protein levels, although the differences between acute and chronic exposure intra-dose groups were not significant (Figure 3B). While no significant association was observed between IL6 and IL8 on day 1 (r = 0.1965, *p* = 0.1489; Figure 3C), the correlation analysis highlighted a statistically significant positive association between IL-6 and IL-8 protein levels at day 5 (r = 0.4388, *p* = 0.0189; Figure 3D), indicating a moderate relationship.

### 2.2. HG Influenced the Transcript Expression of Complement Receptors, Effector Fragments and Regulators

To assess the effect of HG on the main complement targets, we evaluated the expression of transcripts specific for *C3aR1* and *C5aR1* receptors as well as *C3*, *C5*, *C9* effector fragments and *CFH, CD55, CD59* regulators, under acute and chronic challenges.

**Figure 4:**
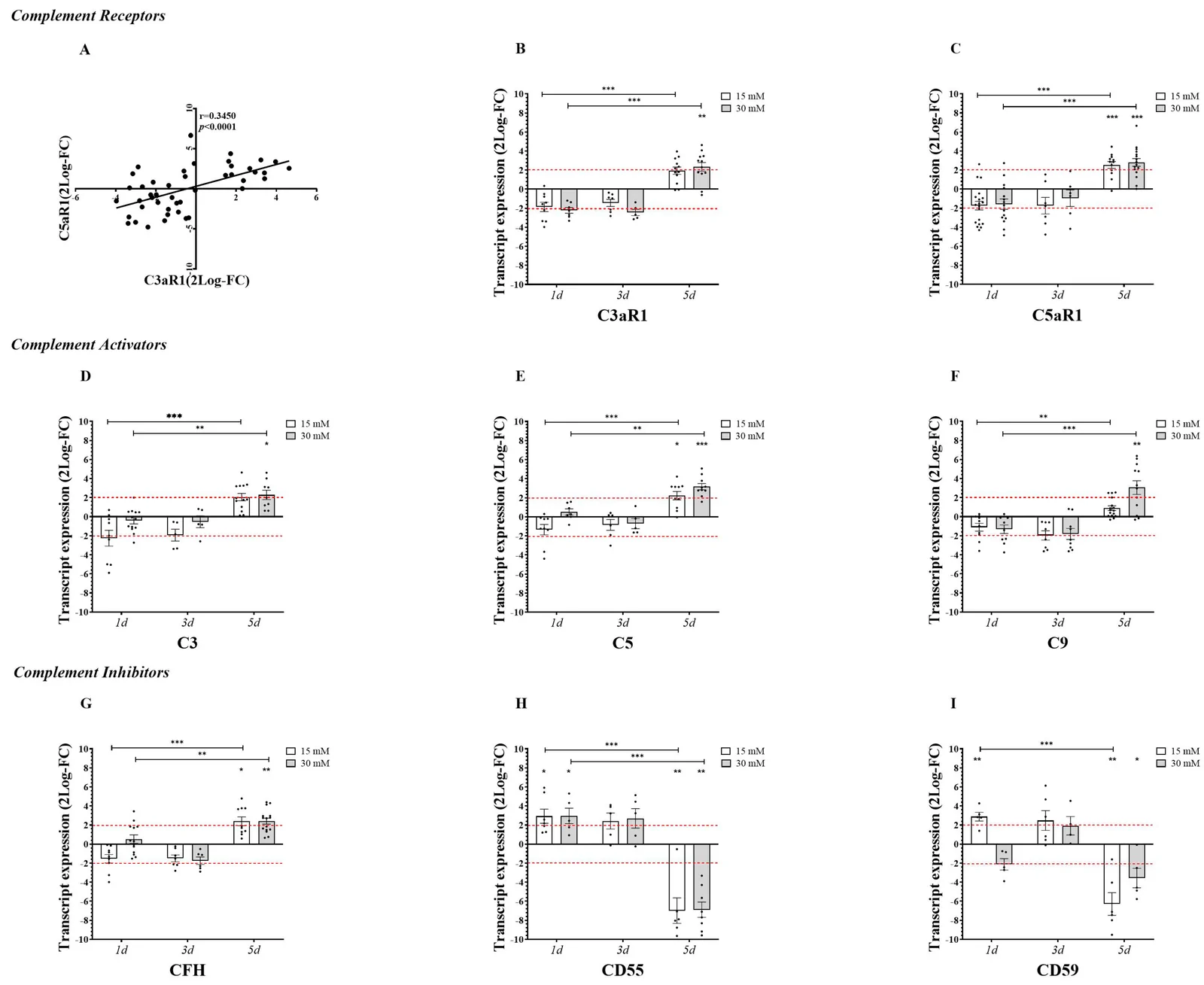
HG exposure modulated the transcript expression of complement mediators. (**A**) Scatterplot displaying a slight positive correlation between *C3aR1* and *C5aR1* after 15 mM HG exposure for all time points investigated. Bar graphs show the transcript expression of complement receptors *C3aR1* (**B**) and *C5aR1* (**C**), complement activators *C3* (**D**), *C5* (**E**) and *C9* (**F**), and finally complement modulators *CFH* (**G**), *CD55* (**H**) and *CD59* (**I**) in CECs exposed to 15 mM HG (white bars) or 30 mM HG (grey bars) over 1, 3 and 5 days. Expression ratios were calculated relative to untreated control cells and data are shown as mean ± SEM. Pearson correlation coefficient (r) and significance are shown inside panel (**A**). Statistical comparisons were performed with 2-way ANOVA Dunnett’s post hoc analysis for time-point and group comparisons (**B**–**I**), and significance were * *p* < 0.05; ** *p* < 0.01; *** *p* < 0.001. The red dashed lines were included as an additional stricter cutoff identifying biologically relevant changes. Legend: *C3aR1*, Complement C3a Receptor 1; *C5aR1*, Complement C5a Receptor 1; *C3*, Complement component 3; *C5*, Complement component 5; *C9*, Complement component 9; *CFH*, Complement Factor H; *CD55*, Cluster of Differentiation 55; *CD59*, Cluster of Differentiation 59.

As shown in Figure 4A, the bar-graph displays a statistically significant positive association between *C3aR1* and *C5aR1* transcript levels across all time points (r = 0.3450, *p* < 0.0001), although the linear association was relatively weak. Under acute challenge and two exposures, a trend to a downregulation of transcript expression was observed for *C3aR1* (Figure 4B) and *C5aR1* (Figure 4C); the same trend was observed for *C3*, *C5* and *C9* effectors (Figure 4D–F) and *CFH* regulator molecule (Figure 4G). On the contrary, an upregulation was observed for *CD55* (Figure 4H) and *CD59* (Figure 4I) from day 1 to day 3, although *CD59* showed an opposite downregulation trend at 30 mM HG on day 1. Under chronic conditions, the complement profile reversed and particularly *C3aR1* was significantly upregulated at 15 mM HG and *C5aR1* was significantly upregulated at both 15 mM HG and 30 mM HG. With respect to effector targets, transcripts for *C3, C5* and *C9* fragments were upregulated at both 15 mM and 30 mM HG, with higher expression for *C5* and *C9* at 30 mM with respect to 15 mM HG. Finally, transcript expression of *CFH* regulator was deregulated at both 15 mM and 30 mM at day 1 and day 3, while upregulated at both HG doses at day 5. Lastly, a downregulation of transcript expression was observed for *CD55* and *CD59* at both 15 mM and 30 mM challenges, while trends to increase were observed at day 1 and day 3 for both 15 mM and 30 mM HG.

**Figure 5:**
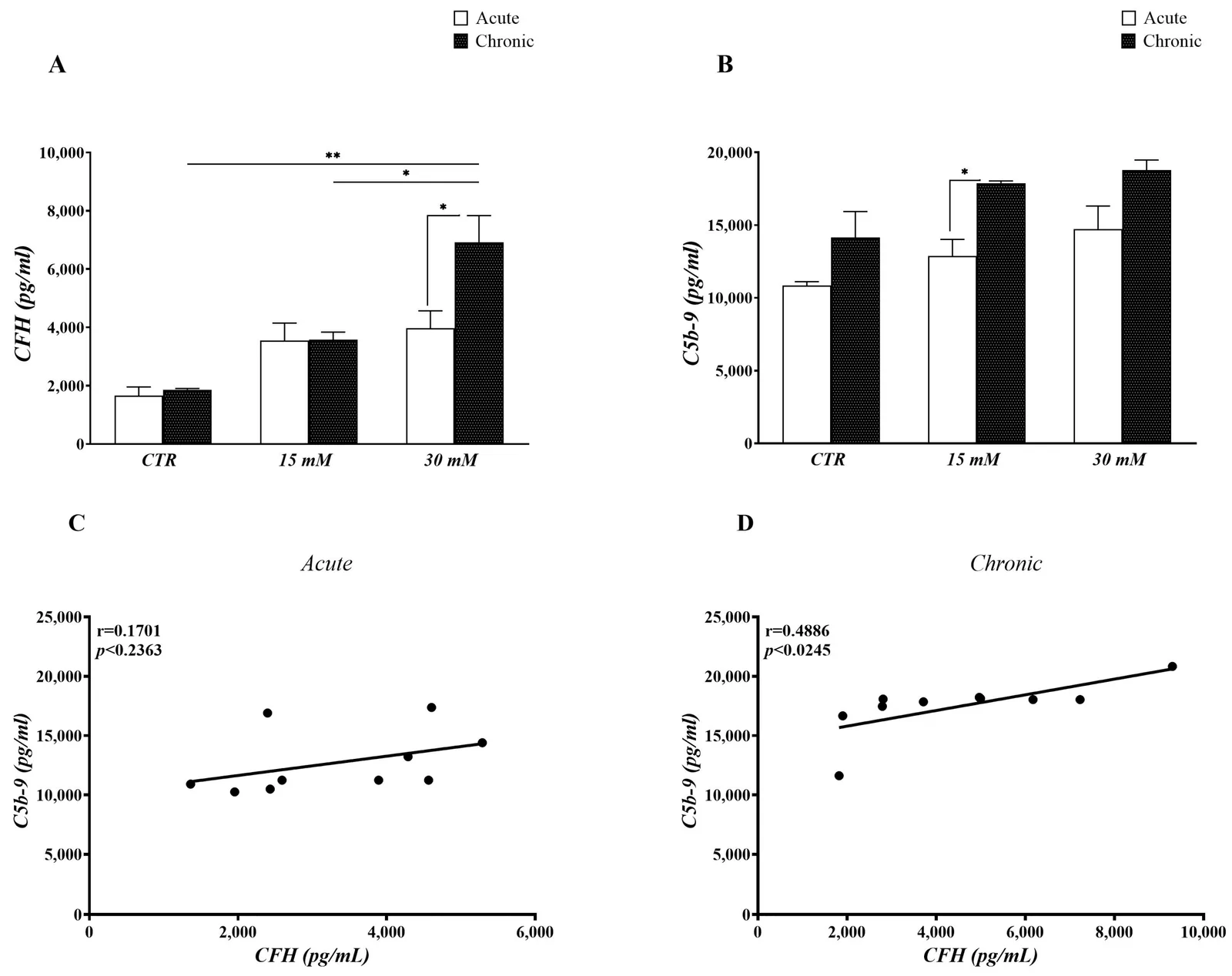
Acute and chronic HG exposure increased CFH and C5b-9 protein levels. CFH (**A**) and C5b-9 (**B**) protein levels were measured respectively in conditioned media and cell pellet from 15 mM HG or 30 mM HG upon acute (day 1) and chronic (day 5) exposure, in comparison to control cells (CTR; ELISA). Data are expressed as mean ± SEM (pg/mL) from four independent experiments performed in triplicate. Two-way ANOVA followed by a Tukey–Kramer post hoc test analysis (glucose vs. timing) provided the statistical differences shown in the panels by asterisk (* *p* < 0.05; ** *p* < 0.01). (**C**,**D**) Scatterplot displaying the relationship between CFH and C5b-9 protein levels after acute (**C**) and chronic (**D**) HG exposure. Pearson correlation coefficient (r) and significance are shown in the panel. Legend: CTR, control cells; CFH, complement factor H; C5b-9, Membrane Attack Complex (MAC).

Both CFH and C5b-9 protein levels were quantified in respectively conditioned media and cell pellets by specific ELISA, showing a similar trend of expression. Precisely, CFH release followed a HG concentration-dependent manner, rising significantly at day 5 under 30 mM HG exposure (Figure 5A). As well, the cell-associated C5b-9 protein accumulated progressively with increasing HG exposure C5b-9 (Figure 5B). Finally, correlation analysis showed no significant association between CFH and C5b-9 after day 1 acute challenge (r = 0.1701, *p* = 0.2363, Figure 5C), while a significant positive correlation was observed at 5 days (r = 0.4886, *p* = 0.0245, Figure 5D).

### 2.3. HG Influenced TLR-2 but Not MyD88 Expression

**Figure 6:**
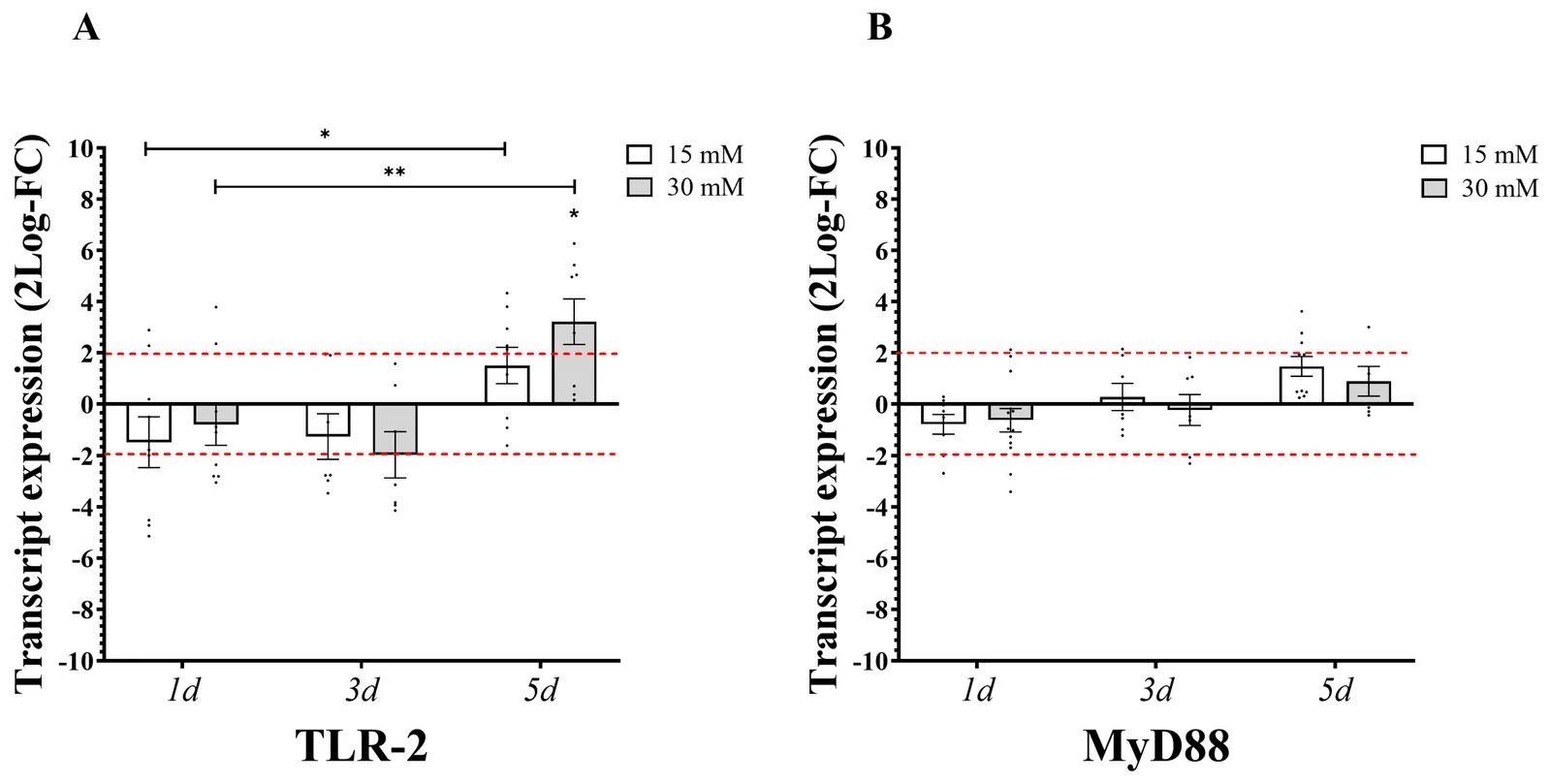
HG-exposed CECs displayed changes in *TLR-2* and *MyD88* transcript expression. Bar graphs showing the 2 log transcript expression specific for *TLR-2* (**A**) and *MyD88* (**B**) in CECs exposed to 15 mM HG (white bars) or 30 mM HG (grey bars) over 1, 3 and 5 days. Expression ratios were calculated relative to unexposed control cells (CTR) and data are shown as mean ± SEM. The red dashed lines were included as an additional stricter cutoff identifying biologically relevant changes. Data were statistically analyzed by 2-way ANOVA and mixed-effects models with Dunnett’s post hoc tests for time-point and group comparisons. Statistical significances are shown by asterisk (* *p* < 0.05; ** *p* < 0.01). Legend: *TLR-2*, Toll-like receptor-2; *MyD88,* Myeloid Differentiation primary response 88.

To evaluate any changes in TLR-2/MyD88 response to HG exposure, we evaluated the transcript expression of *TLR-2* and its common downstream adaptor *MyD88* under acute and chronic HG exposure over time (Figure 6). Under acute challenge and two exposures, a trend to a downregulation of transcript expression was observed for *TLR-2* (Figure 6A) and *MyD88* adaptor molecule (Figure 6B) transcripts were significantly deregulated at 15 mM and 30 mM HG. On the contrary, chronic exposure to 30 mM HG showed a selective *TLR-2* transcript upregulation (Figure 6A) while *MyD88* did not (Figure 6B).

**Figure 7:**
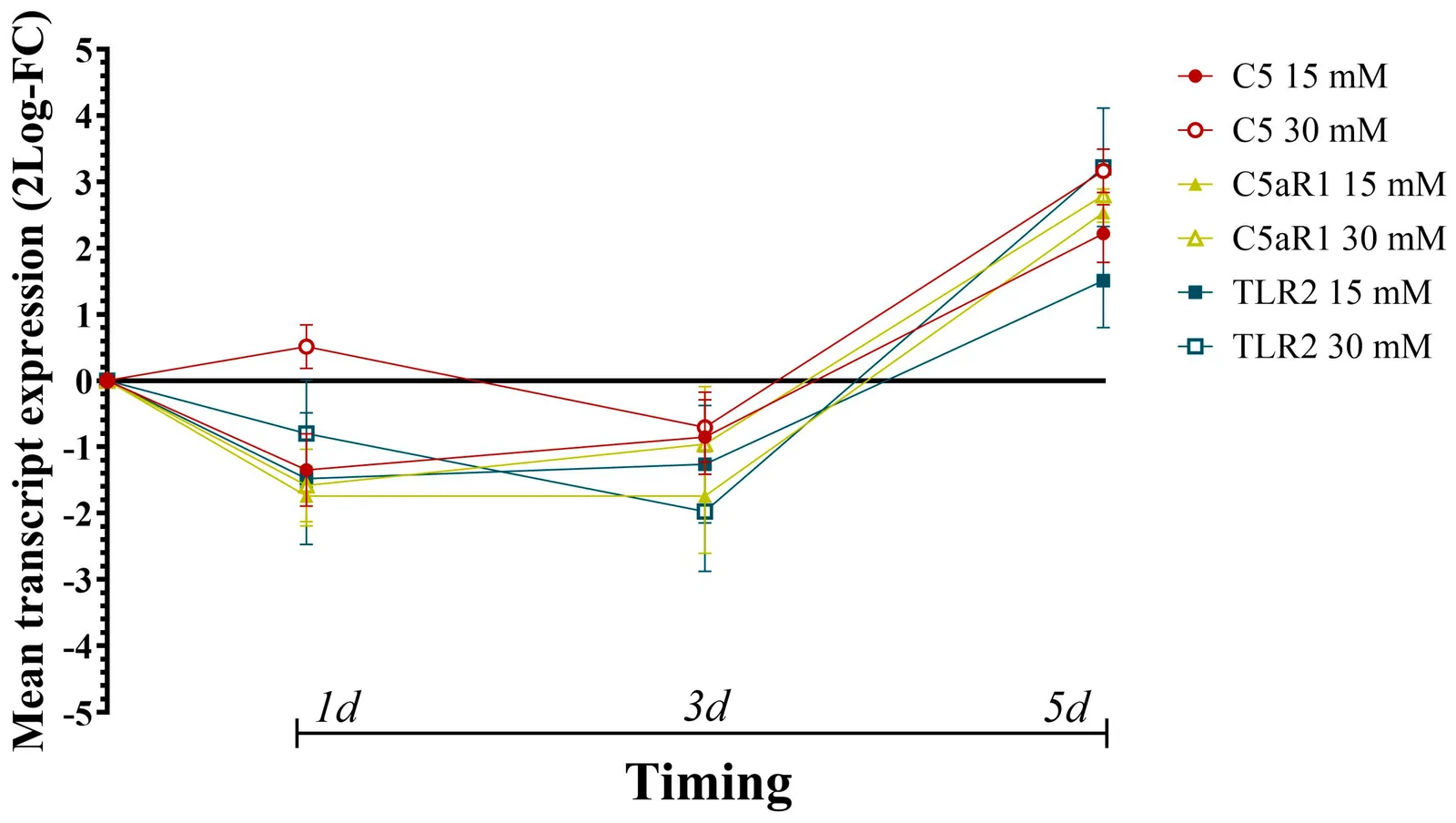
Coordinated expression profiles of *C5, C5aR1* and *TLR-2* in HG-exposed CECs. Connected dot plot showing expression ratios for *C5* (red), *C5aR1* (yellow) and *TLR-2* (blue) upon acute and chronic challenges over time (day 1, day 3 and day 5). Note that only 30 mM HG (open square symbols) showed the highest expressions (over 2 log biochemical threshold) for all transcripts investigated after chronic challenge (day 5) with respect to 15 mM HG. Values (mean ± SEM) are expressed relative to untreated controls. Legend: *C5*, Complement component 5; *C5aR1*, Complement C5a Receptor 1; *TLR-2*, Toll-like receptor-2.

Changes between complement (*C5* and *C5aR1*) and *TLR-2* transcripts across all conditions are shown in Figure 7. A marked upregulation was observed for all targets after chronic stimulation and particularly at 30 mM HG.

### 2.4. Acute and Chronic HG Exposure Influenced Differentially the Main Antioxidant Mediators and Few Epigenetic Targets

**Figure 8:**
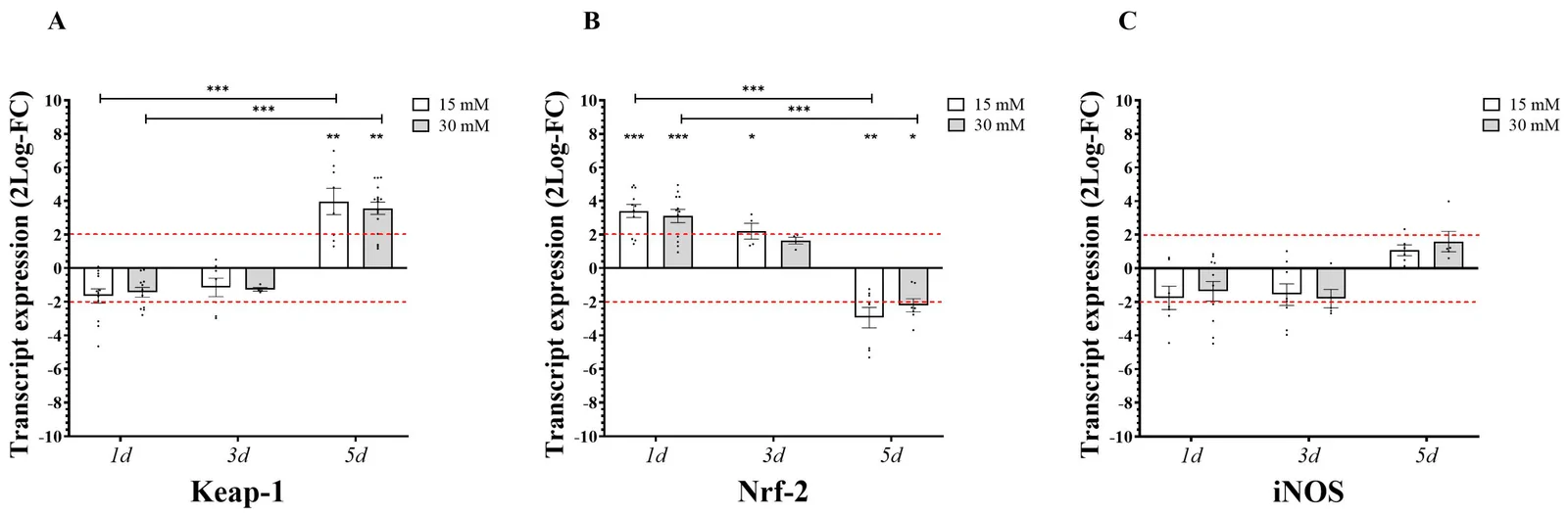
HG-exposed CECs displayed changes in *Keap-1, Nrf-2* and *iNOS* transcript expression. Bar graphs showing the 2 log transcript expression specific for *Keap-1* (**A**), *Nrf-2* (**B**) and *iNOS* (**C**) in CECs exposed to 15 mM HG (white bars) or 30 mM HG (grey bars) over 1, 3 and 5 days. Expression ratios were calculated relative to untreated cells (controls). Data were analyzed via two-way ANOVA and mixed-effects models with Dunnett’s post hoc tests for time-point and group comparisons. Statistical significance was set as * *p* < 0.05; ** *p* < 0.01; *** *p* < 0.001. The red dashed lines were included as an additional stricter cutoff identifying biologically relevant changes. Legend: *Keap-1*, Kelch-like ECH-associated protein 1; *Nrf-2*, Nuclear Factor (erythroid-derived 2)-like 2; *iNOS*, Nitric Oxide (NO) Synthase.

To examine the HG-related cellular antioxidant response, we evaluated the transcript expression of *Keap-1*, *Nrf-2* and *iNOS* under acute and chronic HG exposure conditions. On day 1 and day 3, *Keap-1* transcripts (Figure 8A) were slightly deregulated, while *Nrf-2* transcripts (Figure 8B) were significantly upregulated at both 15 mM and 30 mM HG. On day 5, *Keap-1* transcripts were significantly upregulated at both concentrations, while an opposite effect was observed for *Nrf-2* transcript expression. On day 1 and day 3, *iNOS* transcripts (Figure 8C) were slightly deregulated at both concentrations, while at day 5 an upward trend was observed for *iNOS* transcripts, for both 15 mM and 30 mM HG.

**Figure 9:**
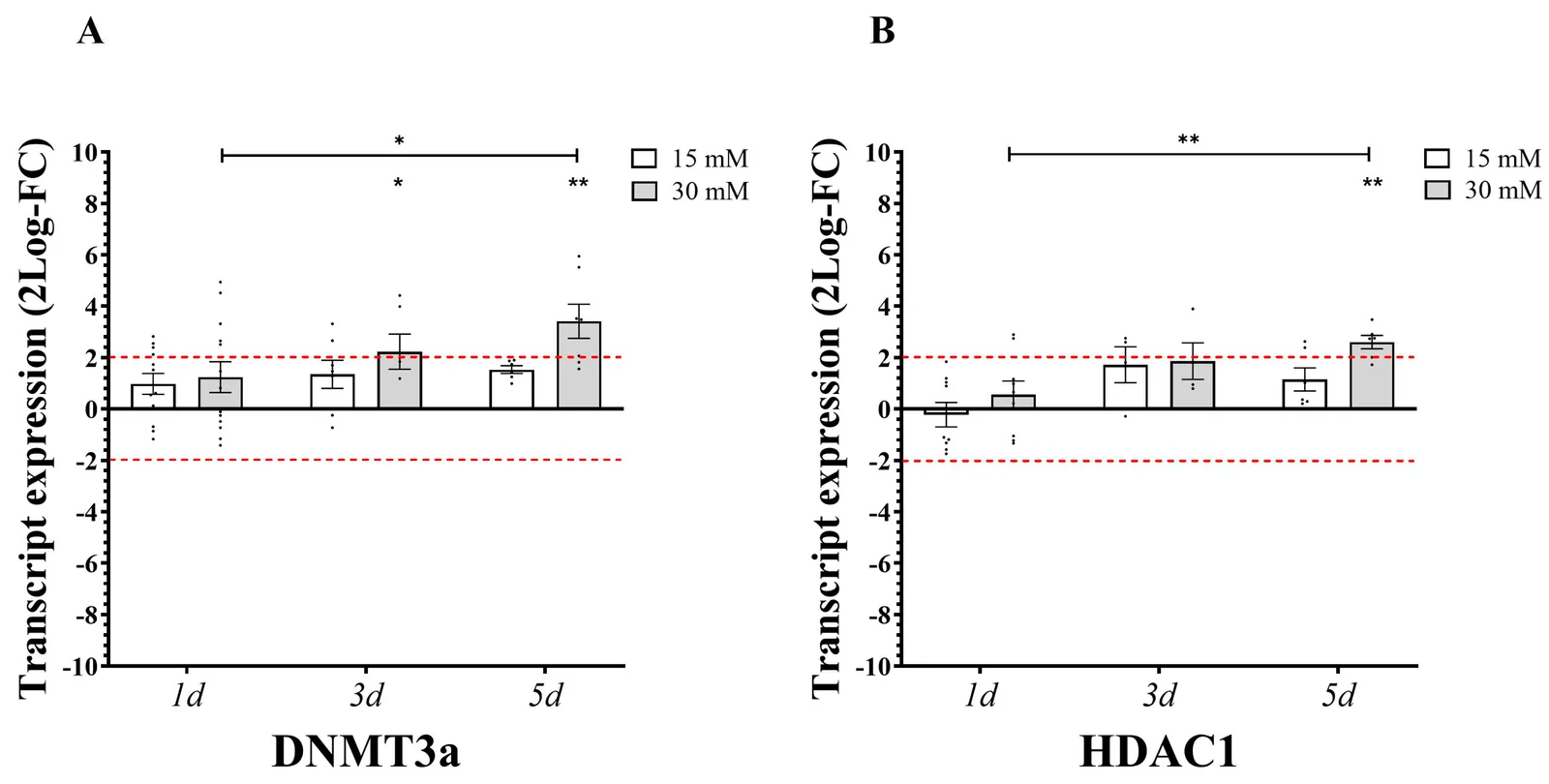
*DNMT3a* and *HDAC1* expression in HG-exposed CECs. Bar graphs show the transcript expression ratios specific for *DNMT3a* (**A**) and *HDAC1* (**B**) in CECs exposed to 15 mM HG (white bars) or 30 mM HG (grey bars) over 1, 3 and 5 days. Expression ratios were calculated relative to untreated control cells. Data were analyzed via two-way ANOVA and mixed-effects models with Dunnett’s post hoc tests for time-point and group comparisons, respectively. Statistical significance was set as * *p* < 0.05 and ** *p* < 0.01. The red dashed lines were included as an additional stricter cutoff identifying biologically relevant changes. Legend: *DNMT3a*, DNA (cytosine-5)-Methyltransferase 3A 1; *HDAC1*, Histone Deacetylase 1.

To investigate potential changes at the DNA transcription level, two DNA enzymes (*DNMT3a* and *HDAC1*) were investigated at transcriptional level. From day 1 to day 5, both *DNMT3a* (Figure 9A) and *HDAC1* (Figure 9B) showed a progressive upregulation of transcript expression. Specifically, *DNMT3a* transcription was significantly high at 15 mM HG on both day 3 and day 5, whereas *HDAC1* transcription increased significantly only to 15 mM HG on day 5.

### 2.5. HG Triggered the Expression Profiles of β-NGF and BDNF

**Figure 10:**
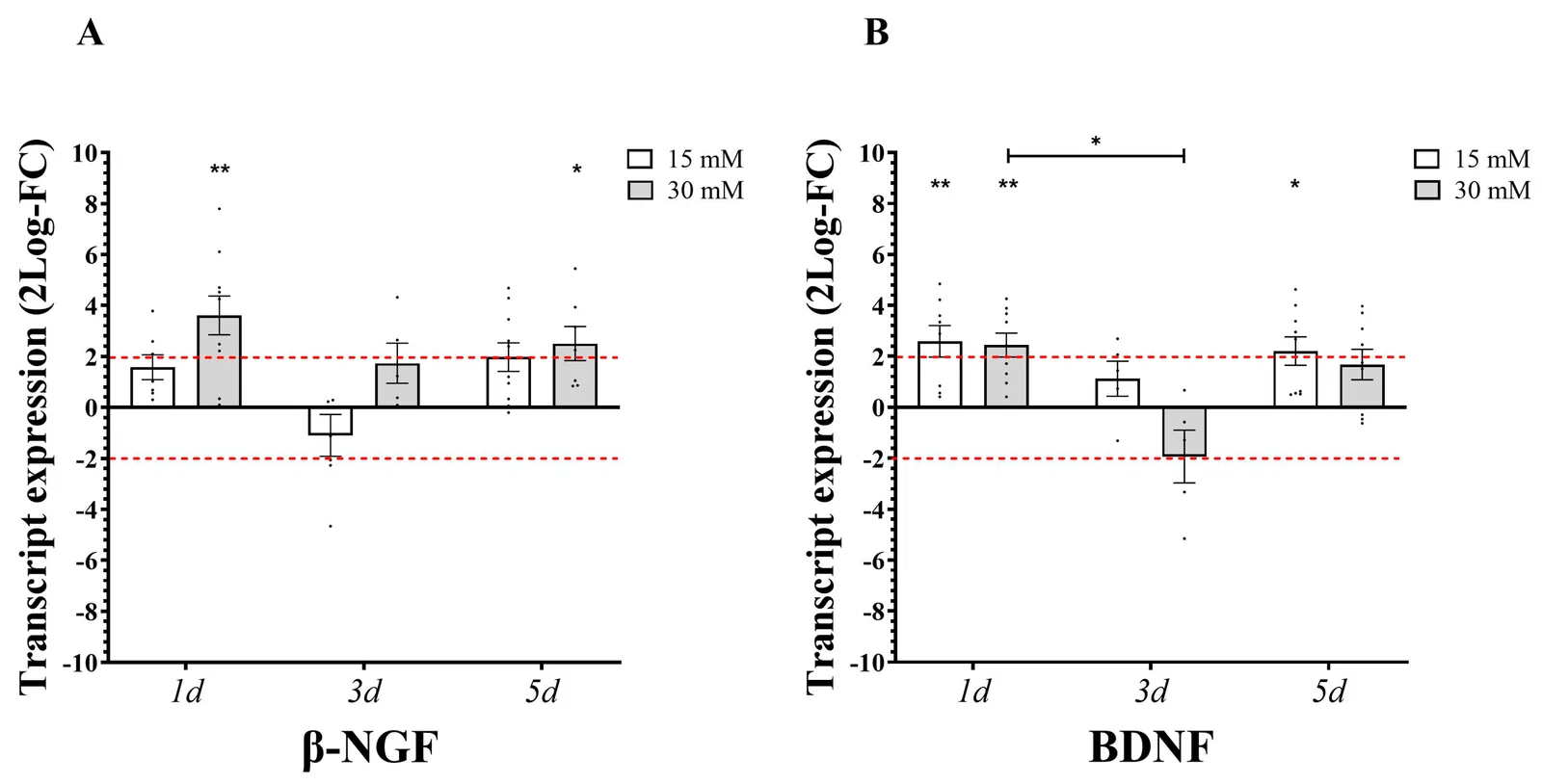
HG-exposed CECs displayed changes in *β-NGF* and *BDNF* transcript expression. Bar graphs show the transcript expression specific for *β-NGF* (**A**) and *BDNF* (**B**) in CECs exposed to 15 mM HG (white bars) or 30 mM HG (grey bars) over 1, 3 and 5 days. Expression ratios were calculated relative to untreated cells (controls). Data were analyzed by two-way ANOVA and mixed-effects models with Dunnett’s post hoc tests for time-point and group comparisons, respectively. Statistical significance was set as * *p* < 0.05 and ** *p* < 0.01. The red dashed lines were included as an additional stricter cutoff identifying biologically relevant changes. Legend: *β-NGF*, β-Nerve Growth Factor; *BDNF*, Brain Derived Neurotrophic Factor.

To evaluate neurotrophic release, we analyzed the transcript expression specific for *β-NGF* and *BDNF* under acute and chronic HG over time. On day 1, *β-NGF* (Figure 10A) was significantly upregulated selectively at 30 mM HG exposure, while *BDNF* (Figure 10B) increased under both 15 mM and 30 mM HG exposures. On the contrary, a significant reduction was observed by day 3 for both neurotrophic factors. A partial recovery was observed on day 5: *β-NGF* transcripts were significantly upregulated at 30 mM HG exposure, while *BDNF* increased significantly at 15 mM HG exposure.

## 3. Discussion

Herein the study provides a comprehensive characterization of the molecular and biochemical response of primary human corneal epithelial cells (CECs) to acute (day 1) and prolonged (day 3, day 5) High Glucose (HG) exposure. By combining transcriptional profiling with biochemical assessment of a few selected mediators (inflammatory and complement mediators, innate receptors, oxidative stressors, DNA enzymes and neurotrophins), we were able to investigate the cellular response at both gene expression and protein levels. This integrated approach revealed a time- and dose-dependent response, characterized by an initial adaptive phase followed by progressive molecular and biochemical dysregulation under prolonged HG exposure. Acute (day 1) and chronic (day 3, day 5) 15 mM and 30 mM HG exposures were tested for key mediators known to exert critical changes at the corneal epithelia, as reported by previous studies.

Corroborating findings indicate that metabolic disorders, such as diabetes, can affect corneal epithelial cells, as observed by the developing of keratopathy in diabetic subjects with delayed corneal wound healing, reduced corneal sensitivity and recurrent corneal epithelial erosion [2]. Neurotrophic changes and superficial punctate keratopathy were also reported, potentially compromising vision [3]. The mechanism underlying these epithelial changes is still controversial. The herein investigated major tasks representative of the physio-pathological changes at the ocular surface are inflammation (mainly *IL-8*, *IL-6*), complement actors (*C3*/*C3R1*, *C5*/*C5R1*, *C9* and *C5b-9* complex), modulators (*CFH* and *CD55*/*CD59*), Toll-like receptors (mainly *TLR-2*/*MyD88*), oxidative stressors (mainly *Keap-1*, *Nrf-2*, *iNOS*), DNA-binding enzymes (mainly *DNMT3A* and *HDAC1*) and neurotrophins (mainly *β-NGF* and *BDNF*), showing tidy crosstalks and regulation aspects [1,2]. Growing evidence suggests that alterations in the expression of some mediators belonging to these clusters can play a key pathogenic role at the ocular surface, contributing to tissue damage with recruitment of inflammatory mediators that might be exacerbated by diabetes [10]. Previous studies from cultured corneal epithelial cells demonstrated that only chronic HG stress leads to mitochondrial dysfunction, with hyperglycemia-induced loss of spare respiratory capacity reducing the ability of corneal epithelial cells to respond to subsequent stress [5].

First, the analysis of cell viability and metabolic activity showed that HG exposure triggered an initial compensatory/proliferation response followed by loss of cellularity under prolonged HG exposure, that does not imply a severe loss of viability and metabolic activity of adherent cells that appeared alive and likewise metabolically active.

With respect to the pro-inflammatory pattern, the absence of *p65NFκB*-*IL-1β* linkage across all tested conditions would imply that *IL-1β* induction proceeds largely through NFκB-independent mechanisms. Considering that in corneal epithelial cells High Glucose can trigger the activation of NLRP3 inflammasome, that is associated with ROS generation and advanced end-product glycation, a consequence of diabetic hyperglycemia at ocular surface can be prospected [15,16,17,18]. Indeed, the *IL-1β* transcripts’ increase across all time points with no dose-dependent fashion would suggest a non-linear transcriptional response to High Glucose [19]. The two major acute phase cytokines *IL-6* and *IL-8* were transcriptionally upregulated upon acute HG challenge, indicating an early activation of the pro-inflammatory axis [19]. As key findings, *IL-6* transcript and protein results were broadly consistent, an effect not observed for *IL-8*. Initially *IL-6* and *IL-8* transcripts followed the same transcriptional pattern although *IL-8* transcripts declined at higher HG dose under chronic conditions. Despite a marked decrease in *IL-8* transcripts at 30 mM HG after 5 days, IL-8 protein levels continued to accumulate in the conditioned media, suggesting that transcript degradation might proceed independently of secretory activity, reflecting the post-transcriptional regulation of IL-8 which transcript stability might be ruled by AU-rich elements (AREs) and suggesting that HG suppressed *IL-8* transcripts in a cell-type-specific manner [20]. To support, a reduced *IL-8* expression was reported in non-endothelial cells but not in aortic endothelial cells showing an opposite effect [21].

In diabetes, central effectors of innate immunity are the complement system and the Toll-like receptors (TLR) [8]. The complement findings, which included activators, effectors and inhibitors, can be viewed as the main intriguing aspect of this study. Complement dysregulation upon hyperglycemia has been well documented in retinal cells, in consideration of the frequent diabetic retinopathy, but not in corneal epithelial ones [22,23,24,25]. The acute complement response mediated by 15 mM HG exposure could reflect a coordinated homeostatic strategy, as suggested by the downregulation of *C3aR1* and *C5aR1* receptors as well as *C3*, *C5* and *CFH* mediators, and the upregulation of membrane-bound *CD55* and *CD59* regulators. By contrary, 30 mM HG exposure impaired this balance as observed by the significant downregulation for *CD59* and a trend to a downregulation for *CD55* [26]. Since both *CD55* (DAF) and *CD59* are GPI-anchored proteins in charge for protecting the host cell from autologous complement attack, this HG-dependent reduction might be explained as a potential involvement in the glycation-mediated damage, resulting in a loss of protection and increased susceptibility to damage of ocular surface [27,28]. The fact that HG-mediated chronic phase promoted an increase in *C3aR1* and *C5aR1* transcripts while *CD55* and *CD59* transcripts were completely downregulated sustains the above prospected conclusion. On the contrary, the observation of HG-dependent *CFH* release following severe (30 mM HG) and chronic (day 5) exposure would suggest the developing of potential restorative cellular attempts to counteract complement-mediated damage, an aspect that can be translated to in vivo contest. Together, these differential complement pattern expressions might suggest a shift from acute control to a chronic loss of regulatory control, reflecting the impairments of complement under sustained hyperglycemia [29]. This observation is in line with previous studies from diabetic non-ocular tissues showing that chronic hyperglycemia promotes complement dysregulation through progressive downregulation of proper protective regulators [10,11]. Since *CFH* transcriptional data were corroborated by biochemical ones, an impaired extracellular compensatory response should be considered, as reported in diabetic retinopathy [30]. As well, the *C5b-9* protein increase upon HG exposure might suggest the activation of the complement terminal pathway. Interesting, the positive relationship between CFH and MAC/C5b-9 upon chronic exposure would suggest a complement-mediated damage overcoming cellular defense and sustaining a chronic inflamed state amplified by *p65NFκB* activation [31]. To better explain, the chronic HG-mediated CFH upregulation might represent an insufficient compensatory response, unable to prevent the progressive accumulation of MAC/C5b-9 on the cell surface. Considering the data on cell viability, it might be possible that C5b-9 does not cause directly cell lysis but works in a sub-lytic regime sufficient to amplify IL-6 and IL-8 release and sustain the pro-inflammatory loop, as previously reported for retinal pigment epithelial cells [31]. Certainly, the inhibition of MAC/C5b-9 would represent an interesting point for developing focused therapeutic strategies. ELISA analysis corroborated the transcription ones on pro-inflammatory IL-6 and IL-8 cytokines and MAC/C5b-9 and CFH complement proteins.

The crosstalk between complement system and innate receptor (particularly *TLR-2/MyD88*) plays crucial roles in the pathogenesis of diabetes mellitus (Type 1 and Type 2) and its complications [11]. As stated, TLRs serve as a key mediator of innate immune pattern recognition and work in concert with complement axis. Upon acute challenge, *TLR-2* and *MyD88* adaptor molecule transcripts were significantly deregulated at all doses tested, indicating a general dampening of *TLR* signaling during the acute phase. Chronic exposure of 30 mM HG showed a selective *TLR-2* transcript upregulation in the absence of *MyD88* regulation, positioning *TLR-2* as a dynamic modulator of innate immune response against hyperglycemia, herein mimicked by HG exposure. Another point of interest is represented by the convergence of *C5/C5aR1* and *TLR-2* expression under 30 mM HG chronic challenge. As well documented, the molecular crosstalk between *C5aR1* and *TLR-2* has been previously reported, and particularly *C5aR1* signaling was found to potentiate *TLR-2*-mediated response [32,33]. In our in vitro study, *C5*/*C5aR1* and *TLR-2*/*MyD88* crosstalk might be hypothesized, providing additional explanations in diabetic corneal keratopathy and other hyperglycemia-driven ocular diseases [34]. This crosstalk might be amplified by *p65NFκB* and *C3*, which were upregulated in our in vitro model and other studies showing that *C3* stimulates the *TLR-2/NFκB* signaling pathway [34]. To support, previous studies reported that the crosstalk between *C5a*/*C5aR1* and *TLR-2* axes upon chronic hyperglycemic states can fast the peripheral nerve damage and neuropathic pain [32,33]. Although not fully investigated in this study, these transcript changes might mirror a coordinated shift from an early innate immune-regulated response (complement and TLRs) towards a chronic complex inflammatory one (*IL-6* and *IL-1β*), reflecting the shift from parainflammation to inflammation, as prospected in diabetic corneal keratopathy [35]. The parainflammation is a low-level adaptive immune response that is triggered by long-lasting tissue stress, including hyperglycemia and/or oxidative stress, protecting from cellular damage and restoring tissue homeostasis without causing the clinical symptoms of acute inflammation [35].

Studies in diabetic eyes reported that the increased expression of *Keap-1* can impair *Nrf-2*-mediated antioxidant response, promoting Reactive Oxidative Stress (ROS) accumulation and thereafter inflammation at the ocular surface [2,36]. Our results on *Keap-1*, *Nrf-2* and *iNOS*, known to be key effectors of oxidative stress and nitric oxide synthesis, reveal a temporally delayed but mechanistically important component of HG response. Under acute challenge, *Nrf-2* was significantly upregulated at both concentrations without *Keap-1* expression, suggesting the absence of any feedback control [37]. Under chronic challenge, *Keap-1* was significantly upregulated, while *Nrf-2* was deregulated. At the same time, *iNOS* showed a parallel rising trend with *Keap-1*, an aspect more consistent with the *Keap-1*/*Nrf-2* axis [37,38].

Finally, the investigation of a few selected enzymes with a key role in regulating gene expression, cell survival, and proliferation, revealed an additional intriguing aspect played by HG exposure. A progressive *DNMT3a* and *HDAC1* transcript upregulation was found from day 1 to day 3 and sustained on day 5, with *DNMT3a* rising significantly at 30 mM HG and *HDAC1* peaking at 15 mM HG. This early and sustained activation suggests that DNA methylation (*DNMT3a*) and histone deacetylation (*HDAC1)* programs cooperate under HG stress, potentially locking into transcriptional changes through epigenetic mechanisms, as observed in case of the metabolic memory evidence in retinal endothelial cells having HG-driven *HDAC1* activity and *DNMT3a* expression [39,40]. These findings might suggest a time-dependent induction of transcriptional silencing mechanisms, driving the epigenetic consolidation of chronic hyperglycemic response.

The neuropathic involvement is well documented in diabetes, particularly at the corneal epithelial autocrine and paracrine levels [41,42]. Precisely, *β-NGF* and *BDNF* are known to influence energy metabolism, insulin sensitivity and the pathogenesis of diabetes, serving as a key link between neurological complications and metabolic disorders [41,42]. Herein, the observation that the main neurotrophic factors *β-NGF* and *BDNF* increased on day 1, decreased on day 3 and recovered on day 5, with a higher rise at 30 mM HG for *β-NGF* and 15 mM HG for *BDNF*, might suggest a dynamic route between neuroprotection and neurodegeneration. Due to the role of β-NGF in preserving nerve integrity, this fluctuating pattern highlights the metabolic strain of the hyperglycemic corneal epithelium, underlying the neuropathy and impaired healing that define diabetic keratopathy [41,42]. Moreover, this route might be explained with an initial compensatory neuroprotective response, a time of non-response and finally another attempt to recover the neurotrophic route [41,42]. This temporal pattern of neurotrophic expression might also be a dynamic adaptive response under sustained hyperglycemia, as reported in previous studies [41,42].

This 5-day model, which does not fully replicate early changes or the long term of exposure typical of diabetic ocular surface (even stabilized) and associated corneal keratopathy, has some limitations: i. the missing of global proteomic vs. functional assays across all molecular clusters herein investigated; ii. the neutralizing aspects related to the main targets herein described; and iii. the analysis of post-transcriptional regulations of mediators involved in chronic hyperglycemia remains an open question as representing an additional clinical aspect of relevance for supporting the transcriptional findings. Certainly, the validation in ex vivo models might better understand whether systemic hyperglycemia activates all these patterns in corneal epithelial cells, and whether this activation follows a temporally organized, concentration-dependent pattern. Although representing another aspect to study, the inclusion of parallel osmotic equivalent (Mannitol) tests was only devoted to verifying the number of viable cells and the strengthening of metabolic rather than osmotic effects. These preliminary studies showed that CECs exposed to 15 mM and 30 mM Mannitol did not show consistent changes in *p65NFkB*. To exclude hyperosmolarity as a confounding factor, we assessed the transcriptional profiles of key inflammatory, immune, stress and redox mediators following mannitol treatment to match HG osmolarity. These results suggest that mannitol might not exert a meaningful transcriptional effect, likely supporting the fact that the effects on HG-exposed CECs, herein described, are driven by metabolic mechanisms rather than hyperosmolarity.

Taken together, up to 70% diabetic subjects suffer from severe diabetes-related manifesting including those at the ocular surface, two-fold higher than diabetic retinopathy, which can slowly evolve in corneal disorders [14]. Understanding changes occurring upon acute and chronic in vitro exposure might better comprehend the effects of HG exposure, identify some local mediators and suggest experimental approaches to counteract cellular and microenvironment changes. Current therapies for diabetic keratopathy are supportive and centering on the prevention of infection as well as promoting an optimal healing environment, not considering the stress coming from fluctuation in systemic hyperglycemia [2]. Efforts for understanding cellular and molecular mechanisms at the ocular surface are usually focused on understanding how to preserve corneal epithelial homeostasis from any kind of stressor, to counteract deleterious effects by developing potential therapeutic agents capable of promoting corneal homeostasis, re-epithelialization and integrity [2].

## 4. Materials and Methods

### 4.1. Cell Cultures and Analytical Reagents

Primary human corneal epithelial cells (CECs; 3 batches, each one containing more than 1 × 10^6^ viable cells) were purchased from Innoprot (P10871; Elexalde Derio, Spain) and limited expansion passages (0 to 4th) were performed before in vitro stimulations that occurred between 5th and 8th for experiments, according to a standardized procedure. Since primary cells were purchased from a cell facility supplier, the study was approved by the intramural scientific committee (IRCCS-Fondazione Bietti; Rome, Italy). For routine purpose: plasticware were purchased from Starlab (Milan, Italy) and analytic grade reagents were from Euroclone (Milan, Italy) or ICN Biochemicals (Costa Mesa, CA, USA). For culturing purposes: sterile tissue culture plasticware was obtained from NUNC (Roskilde, Denmark); complete Corneal Epithelial Cell Medium (CECM; P60131-SF; Innoprot). The media containing standard 5 mM glucose was supplemented with Corneal Epithelial Cell Growth Supplement-2, 10% Fetal Bovine Serum (FBS), 1 mM Glutamine and 1% pen/strep cocktail, all provided by Innoprot. Hank’s Balanced Sodium Solution (HBSS) and trypsin-EDTA solution were provided by Società Italiana Chimici (Rome, Italy). Daily provided RNAfree MilliQ water from DirectQ (Merck-Millipore, Vimodrone, Milan, Italy) and Phosphate-Buffered Saline (PBS: 10 mM phosphate pH 7.5 and 0.9% saline) were autoclaved and aliquoted for biomolecular purposes.

### 4.2. Cell Culture Management

Experimental in vitro protocol as well as dose- and time exposures were according to previous studies, and schematized in Figure 1A [13,43]. Briefly, the adherent cells were expanded in T21 flasks (0–4th passages; 70% confluence) with complete corneal epithelial medium (5 mM glucose). For stimulation studies, cells were seeded on 24-well (2 × 10^5^) or 96-well (4 × 10^4^) plates depending on experimental setting. Monolayers (95–99% confluent CECs) were exposed to single or repeated,15 mM or 30 mM HG supplemented media, every 2 days from first stimulation, and harvested at day 1 (single/acute challenge) or at days 3 and 5 (repeated/chronic challenge). Mannitol was used as osmotic control and run in parallel as untreated cells. After 24 h from last stimulation, conditioned media were collected and cell monolayers were washed in HBSS and harvested by trypsin solution followed by neutralization with FCS/HBSS. Sampling times included: day 1 (single/acute), day 3 (repeated) and day 5 (multiple/chronic).

### 4.3. Conditioned Media Collection, Monolayer Harvesting and Assessment

Unexposed cells (control cells) and exposed cells were carried out for each experimental plate set. After acute or chronic HG exposures, the conditioned media were quickly collected and the monolayers (2 × 10^5^/well from 24-well plates) were washed in sterile HBSS (pH7.5) and only adherent cells were directly harvested and extracted in TRIzol (total RNA) or SDS-reduced RIPA buffer (total proteins). Conditioned media were gentle centrifugated (3500 rpm/3 min) to remove death cells and used for ELISA.

For viable cell counting, the trypan blue exclusion test was used to count live (not blue stained nuclei) cells each time point, after dye addition (RS-Blue; 0.05% trypan blue syringe; ALCHIMIA SRL, Ponte San Nicolò, Padova, Italy). Cell viability was assessed by counting the number of viable nuclei at each point under inverted light microscope (Eclipse TS100; Nikon, Tokyo, Japan). Merely 5 squares (well/Optic fields) were selected to analyze the 3 diagonal and 2 left- and right-sided selected areas, using a 10 × 10 ocular grid (×100 magnification). Blue stained cells were excluded from counting as death cells. Metabolic activity was monitored on monolayers (2 × 10^4^/well from 96-well plates) according to a standard procedure (MTT; G4000; Promega, Milan, Italy). Briefly, monolayers were exposed to 10 μL/well MTT Solution [3-(4,5-dimethyl-2-thiazolyl)−2,5-diphenyl-2H-tetrazolium bromide] (Immunological Sciences, Rome, Italy) and left at 37 °C under 5% CO_2_. After 4 h, formazan dissolving agent (100 μL) was added to each well and plates were further incubated for 1 h under the same conditions. The number of living cells, directly correlated to the amount of reduced MTT, was quantified by measuring absorbance at 570 nm using a microplate reader (Sunrise; Tecan, Männedorf, Switzerland).

After specific collection or extraction, total RNA (NaNo program) and total protein (A280 program) amounts were quantified by using the microvolume Nanodrop N1000 Spectrophotometer (NanoDrop N1000, Thermo Fisher Scientific, MA, USA). Values were used for normalization purposes.

### 4.4. Biochemical Analysis

To assess secreted IL-6, IL-8, CFH and C5b-9 as well as cell-associated (C5b-9) proteins, specific ELISA were performed according to standard procedure.

Conditioned media were stabilized with protease inhibitor cocktail (1:200; Pierce Biotechnology, Rockford, IL, USA), centrifuged (15,000 rpm, 4 °C, 10 min) to remove cell debris, and quantified spectrophotometrically (A280; NanoDrop N1000, Thermo Fisher Scientific).

Prewashed monolayers were homogenized by ultra-sonication (Sonics, Newtown, CT, USA) in RIPA buffer (50 mM Tris-Cl pH 7.5, 150 mM NaCl, 5 mM EDTA, 1% Triton X-100, 0.1% SDS, 0.5% sodium deoxycholate, 1 mM PMSF, 1 μg/mL leupeptin). Clear supernatants (13,000 rpm, 4 °C, 20 min) were collected and protein quantified spectrophotometrically.

Analysis by ELISA kits included IL-6 (DY206; R&D Systems, Minneapolis, MN, USA), IL-8/CXCL8 (DY208; R&D Systems, Minneapolis, MN, USA), CFH (ab252359; Abcam, Cambridge, UK) and C5b-9 (EEL236; Thermo Fisher Scientific, Waltham, MA, USA).

All ELISA assays were performed according to manufacturers’ instructions. Absorbance was read at λ450 nm (corrected to λ570 nm) using a 96-well plate reader (Sunrise; Tecan, Männedorf, Switzerland), and protein concentrations were extrapolated from a polynomial 3rd-grade standard curves. Data in the figures were normalized for total protein expression.

### 4.5. Molecular Analysis

Total RNAs were extracted according to the TRIfast technique (Euroclone), treated with DNAseI (AB1709; Ambion Inc., Austin, TX, USA), spectrophotometrically analyzed for 260/280 ratio and RNA quantity (Nanodrop N1000 Spectrophotometer). RNA quality was verified randomly in 1% agarose gel electrophoresis (Promega, Milan, Italy) in a horizontal minicell chamber (Biorad, Hercules, CA, USA). Only samples with a 260/280 ratio > 1.8 were used for cDNA synthesis starting from 1 µg total RNA and using the GoScript standardized procedure (Promega, Madison, WI, USA), including random hexamers and dNTs (Promega, Madison, WI, USA) in a OneCycle programmable thermocycler (LifePRO/BIOER; Euroclone, Milan, Italy). For amplification, 3 μL (target gene) and 1 μL (referring gene) cDNA were amplified with SYBR-green hot-start PCR master mix (Hydra Taq; Biolab; Biocell, Rome, Italy) using the Biorad CFX96™ platform equipped with the CFX Maestro 2.3 DXSE software (Bio-Rad Laboratories, Inc, Hercules, CA, USA). Amplification profile was: one cycle of 95 °C/5 min initial denaturation followed by 35–40 cycles at 95 °C/30 s (denaturation), 58–61 °C/25 s (annealing; TA) and 72 °C/30 s (elongation), followed by fluorescence monitoring at 60–90 °C, 0.01 °C for 0.3 s and further elongation at 72 °C/5 min. Cycle threshold values (Cq) from normalized samples with one melting curve were used for REST analysis (https://www.gene-quantification.de, accessed on 24 July 2026) [44]. Changes in gene expressions at two different glucose concentrations (15 mM and 30 mM) were provided as log2 expression ratio with respect to control (5 mM glucose/medium) referring group for all conditions considered and the H3 house-keeping gene. Primer pairs were synthesized by Eurofin MWG Genomics (www.eurofinsgenomics.eu, accessed on 24 July 2026), at least one intron spanning (Primer3 Input ver. 0.4.0), and sequence are reported in Table 1.

### 4.6. Statistical Analysis

Continuous variables are reported as mean ± SD (text) or mean ± SEM (graphics) and are produced from four independent experiments carried out in triplicate for each time point (well) and including untreated or osmolarity controls. For statistical analysis, data were first analyzed by the Shapiro–Wilk tests to verify the assumption of values coming from a normally distributed population. To identify significant changes in relative real-time RT-PCR, comparisons were performed using one-way ANOVA with Tukey’s post hoc test to compare protein levels between experimental groups and two-way ANOVA followed by Dunnett’s post hoc test to evaluate differences between control and single-stimulation groups across time points. Merely to relative real time RT-PCR data, a stricter cutoff was also applied to identify biologically relevant changes (red dashed lines in the bar-graphs), considering that transcripts with log2FC > 2 or <−2 correspond to respectively >4 or <0.25 fold changes. Statistical significance was set at * *p* < 0.05, ** *p* < 0.01, *** *p* < 0.001 and **** *p* < 0.0001, as indicated in the figures. Relationships between variables were assessed using Pearson’s correlation rho (r) test and statistical significance was set at *p* < 0.05. The magnitude and direction of the linear association were evaluated according to the absolute value and sign of r, considering > 0.700 as a criterion for a strenghten of correlation. Analyses and assembled plotters were performed using Prism 11.0 (GraphPad Software Inc., San Diego, CA, USA).

## 5. Conclusions

Taken together, this in vitro study provides a step forward in understanding some cellular signals that might provide support for developing strategies devoted to preserving epithelial cell integrity and/or counteracting the deleterious effects of hyperglycemia variations, as potentially occurring in diabetic subjects. The findings of this study provide new interesting information about the HG-mediated corneal epithelial cell changes regarding the mediators illustrated above, showing a temporally organized and highly dynamic as well as convergent activation across these multiple axes. While moderate HG exposure appeared to trigger an adaptive complement response, the severe HG exposure upset this protective effect. The acute exposure was characterized by *IL-1β* and *IL-6* upregulation, complement and *TLR-2* deregulation, and the complement inhibitors were upregulated, potentially implying a cellular strategy to limited/targeted innate response. This chronic HG exposure was characterized by a progressive failure of acute-related transcription profile with the appearance of a potential *C5/C5aR1* and *TLR-2* crosstalk, complement regulators and antioxidant factors deregulation and finally a sustained secretion of inflammatory mediators. Translating these cellular outputs into a more complex system, with a focus to diabetic keratopathy, the HG-mediated insults occurring at the corneal surface might not be a passive consequence of metabolic injury but an active and self-sustaining innate immune program strictly dependent on long-lasting hyperglycemia fluctuations. Consequently, this biomolecular profile, the result of acute control and chronic amplification responses, might fulfill a real support to the development of alternative strategies for preserving the corneal integrity or counteracting diabetes-induced corneal worries.

## Figures and Tables

**Table 1:** Primer details. Primer pairs were designed as one intron-spanning specifically to produce an amplicon no longer 250 bps (amplicon size). For and Rev sequences are generated by prime3 (v2.6.0) software according to specific parameters applied to specific mRNA complete sequences (www.ncbi.nlm.nih.gov, nucleotide section).

Genes	Primers Pairs		Accession Number
*Referring genes*			
***H3***	F: GCTTCGAGAGATTCGTCGTT	R: GAAACCTCAGGTCGGTTTTG	**NM_005324**
*Target genes*			
*Oxidative stress and redox*			
***Keap-1***	F: TTCAGCTACACCCTGGAGGA	R: CTTGAAGACAGGGCTGGATG	**BC002417.2**
***Nrf-2***	F: ACACGGTCCACAGCTCATC	R: TGCCTCCAAAGTATGTCAATCA	**BC011558.1**
***iNOS***	F: CCCCTTCAATGGCTGGTACA	R: GTTTCCAGGCCCATTCTCCT	**U31511.1**
*Epigenetic regulation*			
***DNMT3a***	F: GCACTCAAGGGCAGCAGATA	R: TTCCAGGCTTCCAGGGTTAG	**C032392**
***HDAC1***	F: GGGATCGGTTAGGTTGCTTC	R: AGGGCCACAGCTGTCCTCATA	**U50079**
*Complement system*			
***C3aR1***	F: TATGCAAGCTCATCCCCTCCAT	R: TGCACATCACAAAAGCCACCA	**BC020742.1**
***C5aR1***	F: TGGCCTTGGTCATCTTTGCA	R: GATGCTGTACAATGGACGTGAAC	**BC008982.1**
***CFH***	F: TTGCACACAAGATGGATGGTCG	R: CATGTAACTGTGGTCTGCGCTT	**Y00716.1**
***C3***	F: CTCAGCATCAACACACACCC	R: GCAGGAGGAAGTTGACGTTG	**NM_000064.4**
***C5***	F: ATTACTGTGCTTCCTCTGGA	R: TCTGGCACCACTCGTAATGT	**M57729.1**
***C9***	F: AAAAGATGCACCGACGCTGT	R: GGGCTCACTTTCACAATCATCCT	**K02766.1**
***CD55***	F: GGCCGTACAAGTTTTCCCGA	R: TAAGGCTGTTTGAGGGATGCAG	**BT007159.1**
***CD59***	F: TGCGTGTCTCATTACCAAAGC	R: GCTGCCAGAAATGGAGTCAC	**BC001506.2**
*Neurotrophic factors*			
***β-NGF***	F: TGAAGCTGCAGACACTCAGG	R: CACCTCCTTGCCCTTGATGT	**BC126150.1**
***BDNF***	F: CCTTTGAGCCTCCTCTTCTC	R: ACTGTCACAGCTCAGCTC	**M61176.1**
*Pro-inflammatory signaling*			
***IL-6***	F: GACAGCCACTCACCTCTTCA	R: CAGTGCCTCTTTGCTGCTTT	**BC015511**
***IL-8***	F: TCTCTTGGCAGCCTTCCTG	R: TGGGGTGGAAAGGTTTGG	**BC013615.1**
***IL-1β***	F: TCAGCACCTCTCAAGCAGAA	R: TCCACATTCAGCACAGGACT	**BC008678.1**
***p65NFkB***	F: CAGAAGCAGGCTGGAGGTAA	R: GTTAGGCACAGGGACAATGC	**L19067.1**
*Patter recognition receptors*			
***TLR-2***	F: TGATGCTGCCATTCTCATTC	R: CGCAGCTCTCAGATTTACCC	**BC033756.1**
***MyD88***	F: AGCGATGCTTCACAGCCTAC	R: CTCCAGCGACTACTGCCACT	**AF406652.1**

## Data Availability

The original contributions presented in this study are included in the article. Further inquiries can be directed to the corresponding author.
